# We have much more to learn about sodium-glucose cotransporter 2 inhibitors and glucagon-like peptide-1 receptor agonists

**DOI:** 10.1093/ehjcvp/pvaf039

**Published:** 2025-06-17

**Authors:** Stefan Agewall

**Affiliations:** Institute of Clinical Sciences, Karolinska Institute of Danderyd, Stockholm, Sweden

**Figure:**
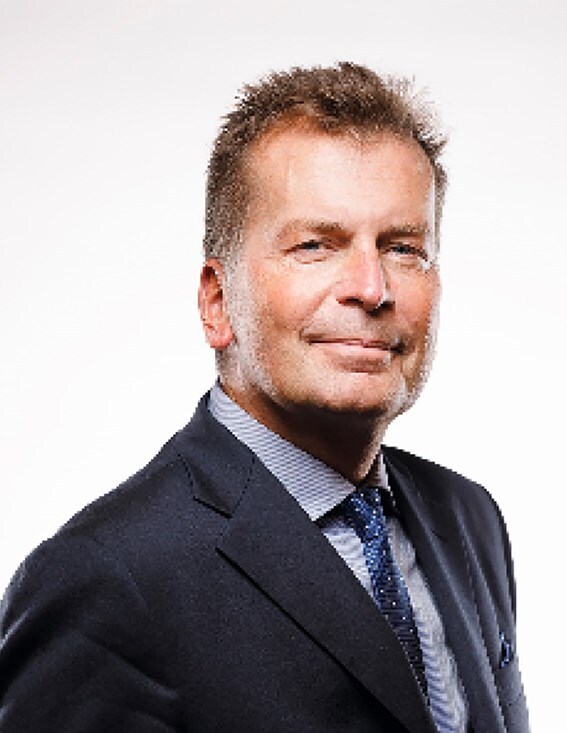


While sodium-glucose cotransporter 2 inhibitors (SGLT2i)^1–4^ and glucagon-like peptide-1 receptor agonists (GLP-1 RA)^3,4^ show cardiovascular benefits, the impact of combining these agents is less clear. Dr Liao and co-workers from Taiwan aimed to evaluate whether adding GLP-1 RA to SGLT2i provides additional benefits in patients with both atherosclerotic cardiovascular disease (ASCVD) and heart failure (HF). They used a database and analysed 96 051 patients with ASCVD and HF patients who initiated GLP-1 RA with SGLT2i or SGLT2i alone.

Sodium-glucose cotransporter 2 inhibitors improve HF outcomes but their effects on acute myocardial infarction (AMI) remains poorly characterized.^5^ Dr Ibrahim *et al.* from USA, aimed to evaluate the 1-year cardiovascular outcomes of SGLT2i among patients with previous AMI regardless of left ventricular ejection fraction, categorized by SGLT2i use. After propensity score matching, 89 554 patients were analysed (44 777 SGLT2-Is users; 44 777 non-users). They report that SGLT2i treatment is associated with improved cardiovascular outcomes in patients with AMI, including reductions in recurrent AMI, all-cause hospitalizations and mortality, and cardiac arrest. The study shows the need for further investigation through prospective randomized clinical trials (RCTs) for confirmation of the results.

Cardiovascular disease and cancer represent significant global health challenges. An overlap between oncology and cardiology is compounded by cancer therapies, which are known to have cardiotoxic effects.^6,7^ SGLT2i, have shown promising cardiovascular benefits but their potential cardioprotective role in cancer patients remains unclear. Dr Madaudo and co-workers from UK, have in a systematic review and meta-analysis evaluated cardiovascular outcomes in cancer patients with type 2 diabetes undergoing chemotherapy with concomitant use of SGLT2i compared with those not using SGLT2i in eleven observational retrospective studies (*n* = 104 327 patients). Their meta-analysis indicated that the use of SGLT2i was associated with a significant reduction in all-cause mortality in actively treated cancer patients with type 2 diabetes. Our study highlights the need for further investigation through prospective RCTs to confirm the efficacy and safety of SGLT2i in attenuating cardiotoxicity and supporting cardiovascular health in oncology settings.

The role of SGLT2i in patients with cardiac amyloidosis (CA) is controversial.^8–10^ Dr D'Amario and co-workers from Italy aimed to evaluate the tolerability and efficacy of SGLT2i in patients with CA. Thirteen observational studies, encompassing 19 227 patients, were included in their meta-analysis. Sodium-glucose cotransporter 2 inhibitors use in patients with CA resulted to be tolerable, as demonstrated by a low absolute cumulative prevalence of both adverse events and was associated with a reduction in the risk of all cause death. Randomized controlled trials are urgently needed to confirm the prognostic improvement associated with their use in this clinical setting.

In a correspondence Dr Hammer from Austria comment on early initiation of SGLT2 inhibitors after AMI.

It is a pleasure to publish review papers lead by Dr Drexel from Austria, about treatment of hyperlipidaemia in patients with different comorbidities.^11^ In this issue of the journal Dr Drexel *et al.* discuss hyperlipidaemia treatment in patients with type I diabetes mellitus. Thus, this specific review summarizes the evidence of lipid-lowering drug classes in reducing cardiovascular risk in patients with type 1 diabetes.

We are also pleased to publish an ‘Expert opinion on the integration of combination therapy into the treatment algorithm for the management of dyslipidaemia’ written by Dr Parhofen and co-workers. Since real-world data shows a large gap between guideline LDL-C goal recommendations and their achievement in clinical practice combination therapy as first-line treatment should be considered to help patients achieve their LDL-C goals.

Also, a third important review within the lipid area is published in this issue. Dr Roustit *et al.* from France present a paper entitled ‘Expert opinion on the integration of combination therapy into the treatment algorithm for the management of dyslipidaemia’. The authors conclude that neither LDL-C nor non-HDL-c demonstrated trial-level surrogacy for predicting treatment effects on mortality and cardiovascular events in statin trials. Although they are relevant biomarkers for the follow-up of patients treated with statins, their reduction does not reliably predict a similar reduction in cardiovascular risk. As such, they should not be used as pivotal evidence in drug trials.

At last in a correspondence Dr Tamargo from Spain presents the hottest new from the American College of Cardiology Meeting 2025.
